# The perceptions on male circumcision as a preventive measure against HIV infection and considerations in scaling up of the services: a qualitative study among police officers in Dar es Salaam, Tanzania

**DOI:** 10.1186/1471-2458-12-529

**Published:** 2012-07-19

**Authors:** Edith AM Tarimo, Joel M Francis, Deodatus Kakoko, Patricia Munseri, Muhammad Bakari, Eric Sandstrom

**Affiliations:** 1 Department of Nursing Management, Muhimbili University of Health and Allied Sciences, Dar es Salaam, Tanzania; 2 National Institute for Medical Research, Mwanza Research Centre, Mwanza, Tanzania; 3 Department of Behavioural Sciences, Muhimbili University of Health and Allied Sciences, Dar es Salaam, Tanzania; 4 Department of Internal Medicine, Muhimbili University of Health and Allied Sciences, Dar es Salaam, Tanzania; 5 Venhälsan, Södersjukhuset, Karolinska Institute, Stockholm, Sweden

**Keywords:** Perception, Police officers, Male circumcision, HIV, Tanzania

## Abstract

**Background:**

In recent randomized controlled trials, male circumcision has been proven to complement the available biomedical interventions in decreasing HIV transmission from infected women to uninfected men. Consequently, Tanzania is striving to scale-up safe medical male circumcision to reduce HIV transmission. However, there is a need to investigate the perceptions of male circumcision in Tanzania using specific populations. The purpose of the present study was to assess the perceptions of male circumcision in a cohort of police officers that also served as a source of volunteers for a phase I/II HIV vaccine (HIVIS-03) trial in Dar es Salaam, Tanzania.

**Methods:**

In-depth interviews with 24 men and 10 women were conducted. Content analysis informed by the socio-ecological model was used to analyze the data.

**Results:**

Informants perceived male circumcision as a health-promoting practice that may prevent HIV transmission and other sexually transmitted infections. They reported male circumcision promotes sexual pleasure, confidence and hygiene or sexual cleanliness. They added that it is a religious ritual and a cultural practice that enhances the recognition of manhood in the community. However, informants were concerned about the cost involved in male circumcision and cleanliness of instruments used in medical and traditional male circumcision. They also expressed confusion about the shame of undergoing circumcision at an advanced age and pain that could emanate after circumcision. The participants advocated for health policies that promote medical male circumcision at childhood, specifically along with the vaccination program.

**Conclusions:**

The perceived benefit of male circumcision as a preventive strategy to HIV and other sexually transmitted infections is important. However, there is a need to ensure that male circumcision is conducted under hygienic conditions. Integrating male circumcision service in the routine childhood vaccination program may increase its coverage at early childhood. The findings from this investigation provide contextual understanding that may assist in scaling-up male circumcision in Tanzania.

## Background

Current Joint United Nations Programme on HIV/AIDS (UNAIDS) statistics show that 68% of total HIV infections occur in sub-Saharan Africa (SSA) [1]. Despite of the availability of already known HIV prevention methods, most new infections continue to occur in this region [1]. Recently, three randomized controlled trials in African countries have demonstrated that medical male circumcision reduces the risk of acquiring sexually transmitted infections (STIs), including HIV, from infected women to uninfected men by about 60% [2-4]. The World Health Organization (WHO) and UNAIDS estimate that approximately 30% of males aged 15 years or older are circumcised globally, and two thirds are Muslims [5]. In addition, the report shows that ethnicity and social- or health-related factors are determinants of male circumcision, and that male circumcision is almost universal in North and most of West Africa. On the contrary, male circumcision is less common in South Africa (SA) [5] where the national HIV prevalence and male circumcision in SA is 18.1% and 35% respectively [6]. In line with the benefits of male circumcision, recently, population-based data from Orange Farm in SA have shown lower HIV prevalence and incidence among circumcised men compared to uncircumcised men [7]. Thus, WHO and UNAIDS have widely recommended the scaling up of male circumcision activities in countries and regions with heterosexual epidemics with high HIV and low male circumcision prevalence [1,8]. It is emphasised that as male circumcision only provides partial protection of male acquisition of HIV, it should not replace other existing biomedical and behavioural interventions. Furthermore, male circumcision has proven to be effective in reducing the risks of penile cancer [9] and cervical cancer in female partners of circumcised men [10,11], urinary tract infections in infants and children [12], ulcerative STIs [13], bacterial vaginosis and trichomonas among female partners of circumcised men [14]. One of the potential challenges in adopting male circumcision in African communities as an HIV intervention strategy may be the lack of awareness that it could minimize risks of HIV transmission.

In Tanzania, the national HIV prevalence is 5.6% [15], and there is great regional heterogeneity with adult HIV prevalence ranging from 1% to 15% [16]. Similarly, the prevalence of male circumcision in Tanzania was estimated to be 70% [6], with some regions having a greater than 95% circumcision rate, while others are as low as 24% [17]. The reasons for the geographical differences could be that male circumcision is influenced by culture, traditions and religion [6]. For regions where male circumcision is mainly done for cultural reasons, about 75% of men are circumcised [18]. In a draft proposal on national strategy for scaling up male circumcision for HIV prevention, the government of the United Republic of Tanzania set a goal of 80% Voluntary Medical Male Circumcision (VMMC) coverage [19]. This strategy prioritizes eight regions of relatively high HIV rates and low male circumcision prevalence, and men aged 10–24 years and 25–34 years are targeted as the primary and secondary priority groups respectively.

There have been many efforts to mitigate the increasing and devastating impact of HIV and AIDS in Tanzania. Initial efforts were directed to HIV prevention as well as reducing the personal and social impact of the epidemic [20]. This was based on Information, Education, and Communication (IEC) campaigns that were regarded as vital in improving people’s knowledge, attitudes and practices on HIV prevention. Additionally, voluntary HIV counselling and testing (VCT) was introduced as a strategy for preventing HIV transmission [17]. The target of the government has been to encourage people who are HIV negative to take definitive steps to avoid becoming infected and for those who are HIV infected to receive the necessary counselling to cope with their status and prolong their life without infecting sexual partners who are negative. Accessibility to antiretroviral therapy (ART) that prolongs life of infected people and reduces the risk of HIV transmission has been a priority, although to date, only 30% of people with advanced HIV infections are able to access ART [15]. For those who are HIV negative, male circumcision has been advocated as an important strategy to complement the existing biomedical prevention methods to reduce HIV transmission from infected women to uninfected men [2-4].

The purpose of this article is therefore to understand the perceptions of male circumcision as a potential strategy against HIV transmission by recruiting members of the Police Force in Dar es Salaam. Members of the police force are heterogeneous as they come from different ethnic groups and religious backgrounds. A previous study showed that the prevalence of HIV in the police force was comparable to that in the general population [21]. Findings from this study will provide an understanding to enable better planning for the scale-up of male circumcision and therefore provision of a holistic HIV prevention package.

The analysis of the data is informed by principles of content analysis guided by socio-ecological model (SEM). The SEM is an approach to health promotion that offers a broad perspective that includes a comprehensive approach integrating multiple levels of influence to impact health behaviour and ultimately health outcomes [22-24]. Improving the health of vulnerable populations may require interventions that target multiple levels of influence [23,25]. In the present study, SEM is used as an analytic tool. The findings are synthesised in the model to acknowledge both personal and environmental influences that will inform the development of targeted interventions [26], specifically the promotion of medical male circumcision.

## Methods

### Setting

The study was conducted within the police stations in Dar es Salaam City, Tanzania. Of the 32 police stations, three stations were purposively and conveniently selected for recruiting study informants.

### Study population

The study was conducted among members of the police force in Dar es Salaam, Tanzania. The police force in Dar es Salaam is comprised of approximately 3,000 police officers [27]. Details of the study population have been presented elsewhere [21,27,28]. The police officers in Dar es Salaam have been involved in HIV-related studies since 1994, and experience shows that they voluntarily consent to participate in HIV-related studies [21]. Between 2006 and 2007, a team of researchers comprised of doctors, nurses and counsellors from Muhimbili University of Health and Allied Sciences (MUHAS), and collaborators from the police force (a doctor and a nurse) conducted sensitization meetings within the 32 police stations. The meetings were meant to increase awareness of current HIV/AIDS prevention strategies and awareness of the need to conduct HIV vaccine trials in Tanzania. Through these meetings, 408 police officers (core group) from the 32 stations agreed to participate in HIV prevention activities within their workplace. The database of all core group members was formed, kept and regularly updated at the MUHAS study site. This core group served as a source of volunteers for a phase I/II HIV vaccine trial in Dar es Salaam (HIVIS-03 study) [29,30]. In this study, the informants were members of the core group, but we excluded those who took part in the HIVIS-03 study because of their extensive exposure to research activities during the trial.

### Sampling

We selected three out of 32 police stations according to availability of a large number of core group members who could fulfil the recruitment criteria. Police officers are often involved in emergency duties outside their ordinary work stations, therefore, the selected stations consisted of members who were most likely to be available. Before approaching the potential informants in their respective stations, the first author sampled the list of core group members from the database at MUHAS. Sampling was done according to age (older and younger), sex (males and females), and religion (Christians, Muslims and other religions). The two collaborators from the police force who were familiar with the study objectives assisted in recruiting the study informants. Thus, purposive sampling was used to recruit the informants in this study. Purposive sampling was meant to provide information-rich cases that will be studied in-depth. There were no strict criteria for determining the sample size, but rather the process of “content saturation” was considered, meaning that data was collected until it became rich, thick and the information started to replicate [31].

### Data collection

We conducted in-depth interviews using a semi-structured question guide. Before data collection, all authors discussed and amended the guide. The guide was used to ensure that the same information was sought from each informant. The research questions focused on: 1) knowledge, beliefs, perceptions and attitudes about male circumcision; 2) perceived reasons regarding why some men have not been circumcised; 3) perceived views of community members towards men who have not been circumcised; and 4) opinions on what can be done to enhance male circumcision practice in Tanzania.

The second author [JF], with a team of trained nurses, conducted the interviews. These nurses had extensive experience in conducting interviews for research purposes. The third author (DK), who is an expert in qualitative methods, reviewed with the nurses the study objectives, interview guide, and the general principles of conducting in-depth interviews: the use of open-ended questions and probes for deeper meaning and understanding of the phenomena; active listening to reflect upon what the informant is saying; note taking; and the information to be collected should be based on principles of saturation. The interviewers stopped enrolling new informants when the information became repetitive and it became clear that there was little to gain by interviewing additional informants. The interviews were audio-recorded and lasted between 30 to 60 minutes. Interviews took place between July and September 2009.

### Data analysis

Analysis started immediately after each interview. JF listened to the audio tapes and wrote a summary of the key findings. The generated summary was used to develop a questionnaire that quantified these qualitative findings [32]. At the same time, two assistants transcribed the audio-recorded material word for word. EAMT listened to 50% of the tapes along with reviewing the transcription to ensure consistency. EAMT and DK independently coded the data on the margins of each transcript. No major differences were noted; however, unclear phrases and words were discussed and negotiated for consensus. A content analysis approach and SEM enhanced data analysis with the levels of SEM guiding the formation of categories. An example of the coding process is presented in Table 1. Quotations are used to represent the informants’ voices in the article.

**Table 1:** An example of coding process

**Codes**	**Sub-categories**	**Category**
Manipulation of penis [Removal of fore skin]	Individual’s knowledge about male circumcision	Intrapersonal level (Individual characteristics that influence behaviour)
Prevention of sexually transmitted diseases		
Hygiene/sexual cleanliness		
Promotion of hygiene		
Religious ritual	Individual’s beliefs towards male circumcision	
Cultural practice		
Transition period	Individual perceptions towards male circumcision	
Increased sexual pleasure		
Confidence during sexual intercourse		
Pain among sexually active men	Individual’s attitudes towards male circumcision	
Interruption of healing process among adults		
Interruption of daily duties among adults		

### Ethical consideration

Ethical clearance was sought and obtained from the MUHAS Institutional Review Board (IRB) with reference No. MU/RP/AEC/Vol.XIII/39. Before data collection, the research permit was obtained from the authorities of each station. Potential informants who fulfilled the recruitment criteria were informed about the objectives of the study and that participation was voluntary. All informants were above 18 years of age. A written informed consent was obtained from each potential informant and informants were notified that they were free to withdraw from the study at any time. The interviewers clarified that the information to be provided was for research purposes and would therefore be strictly anonymous and dealt with confidentially.

## Results

### Informants’ characteristics

A total of 24 men and 10 women participated in the study, and their mean age was 34 and 33 years respectively. The age ranges for men and women were 23–48 and 19–48 years respectively. The majority (85%) had four years of secondary education. Of the total participants, 23 were Christians and the rest were Muslims. Twenty-nine informants identified their ethnic groups (tribes), and they represented 17 different tribes. Of these 29 informants, 11 were originally from regions with high HIV prevalence (above the national prevalence [5.6%]). One informant was a resident of Dar es Salaam. In this qualitative study, the informants’ circumcision status was not investigated.

### Analytical model

The results were synthesised in SEM (Table 2) to provide the informants’ description of their perceptions of male circumcision. In the results, the three levels of SEM were used as categories. The first category was followed with subsequent subcategories as shown below.

**Table 2:** An ecological perspective: levels of influence

**Levels**	**Definition**
Intrapersonal Level	Individual characteristics that influence behavior, such as knowledge, attitudes, beliefs, and personality traits
Interpersonal Level	Interpersonal processes and primary groups, including family, friends, and peers that provide social identity, support, and role definition
Community Level	
Institutional Factors	Rules, regulations, policies, and informal structures, which may constrain or promote recommended behaviors
Community Factors	Social networks and norms, or standards, which exist as formal or informal among individuals, groups, and organizations
Public Policy	Local, state, and federal policies and laws that regulate or support healthy actions and practices for disease prevention, early detection, control, and management

Source: National Cancer Institute. Theory at a glance. Available at http://www.cancer.gov/cancertopics/cancerlibrary/theory.pdf (Cited May 13, 2011).

#### Intrapersonal level (Individual characteristics that influence behaviour)

##### Individuals’ knowledge about male circumcision as a prevention of sexually transmitted infections

Both men and women stated that male circumcision is a procedure of removing or cutting the foreskin of the male sexual organ [penis]. Women in particular related this procedure with a kind of manipulation that exposes the hidden part of the penis:

"“Circumcision as circumcision is the act of removing the outer skin of the male sexual organ in order to expose the head of the penis” (Woman, 27 years, Christian)."

Other informants elaborated that such manipulation enhances hygiene of the private parts. They emphasised that cleanliness of the private parts can prevent sexually transmitted infections. They voiced additional views on how circumcision can contribute to the prevention of sexually transmitted infections from an infected woman to uninfected man. One informant stated:

"“A woman may be infected with syphilis, gonorrhoea; it is very easy to infect a man [uncircumcised] because the fluids [the infectious vaginal fluid] will be retained in the man’s foreskin…” (Man, 37 years, Christian)."

Most of the informants emphasised the importance of circumcision in preventing HIV transmission. They believed that uncircumcised men could easily get HIV from the infected women because of friction between the penis and vagina. They imagined that such friction could cause bruises that can facilitate HIV transmission from infected woman to uninfected man. Nevertheless, they cautioned about the effectiveness of circumcision in preventing HIV transmission. One informant said:

"“It doesn’t mean that HIV transmission cannot occur, it can, but not as quick as when one is uncircumcised; the chances of infection are low” (Man, 40 years, Christian)."

Despite the awareness that male circumcision can minimize chances of HIV transmission, some informants claimed that promotion of male circumcision might facilitate HIV transmission. They suspected that circumcised men might practice unprotected sexual intercourse believing that they are protected. One informant said:

"“If you tell those who are circumcised that they cannot get HIV infection, they will not use a condom. They will even stop all other prevention methods believing that they cannot acquire the HIV infection because they are circumcised. However, if you clarify that the chances of getting infection are low, they will understand” (Man, 35 years, Christian)."

Women emphasised that cleanliness and prevention of diseases are very important during sexual intercourse, and that women tend to disrespect uncircumcised men. One informant narrated:

"“When a woman notes that you [man] are not circumcised, she will disrespect you. This is because the retained powder [old dried seminal fluids] will get into her vagina during sex, and the woman does not know the diseases the powder carries” (Woman, 32 years, Christian)."

In addition, some of the informants stated that during sexual intercourse, an uncircumcised penis needs regular cleaning. Otherwise, the accumulated fluids would have an offensive smell. They added that the foreskin could interfere with penetrative sex. In comparison, they reported that a circumcised penis does not retain the fluids after sexual intercourse. Both men and women felt that a circumcised penis looks clean and good in shape.

##### Individuals’ beliefs towards male circumcision

The informants’ beliefs on circumcision were tied to religious rituals. They expressed their obligation to circumcising young boys to maintain these rituals. Christians referred to documentations regarding circumcision in the Holy Scriptures. They emphasised that after eight days Jesus was circumcised, and that it would be good to adhere to this ritual of circumcising boys while they are still young. Similarly, the Muslims emphasised that uncircumcised men cannot participate in mosque services or in burial ceremonies. They added that for the true Muslims, circumcision is compulsory.

Some of the informants stated that, traditionally, male circumcision is a period of transition for the boys from childhood to adulthood:

"“It is a transition… starting from ten years to fifteen years old. This is the period… For example in the village they prefer to take the boys to the initiation ceremonies [known as jando]” (Man, 37 years, Christian)."

Other informants reported that they circumcise their children based on both religious and traditional principles. One informant said:

"“My culture has influenced me to circumcise my child because I knew that a boy must undergo circumcision. Even in the Bible it is written that boys must be circumcised. Second, I knew if I do not circumcise him, he will feel bad when he becomes an adult” (Woman, 48 years, Christian)."

##### Individuals’ perceptions on male circumcision

The informants perceived that male circumcision could increase sexual pleasure and confidence during penetrative sexual intercourse among adults. Nevertheless, they felt that this kind of pleasure might exacerbate HIV transmission among uninfected men:

"“The chances of HIV transmission are high. During sexual intercourse, psychologically, a circumcised man experiences extra pleasure as compared to the uncircumcised one; and the more pleasure, the more intention to have sex with more women…” (Man, 35 years, Christian)."

Similarly, they added that some women experienced extra sexual pleasure with circumcised men than with uncircumcised ones. One informant stated:

"“Some (women) of us have been reporting that we feel very good when we have sex with a circumcised man…” (Woman, 27 years, Christian)."

##### Individuals’ attitudes towards male circumcision

The informants raised concerns about the age at circumcision. Most of them expressed negative attitude towards male circumcision after childhood. They stated that they would prefer circumcision at early childhood because young boys express less pain than adult men. They reasoned that men have matured vessels. In addition, unlike young boys, men experience sexual desires that lead to erection; the erection may impair the healing process of a circumcised penis:

"“It is difficult for an older man; remember the penis gets bigger and bigger [erection]. Then there is abundant blood supply in the matured vessels, eeh, So they [adults] experience severe pain. I have seen a man in his 40s being circumcised…you can imagine, but for a child it is different” (Woman, 32 years, Christian)."

Most informants supported that it is not desirable for a sexually active man to undergo circumcision. They expressed their worry that when a freshly circumcised man is in need of having penetrative sexual intercourse, the stitches might open up. They emphasised that erection of the penis may interrupt the healing process. For similar reasons, they viewed that uncircumcised men may hesitate to be circumcised. They related this worry to the perception that men establish sexual relationships and start practicing sexual intercourse early. Therefore, undergoing circumcision at older age may not be a desirable decision. However, they stated that if circumcision is compulsory, then their privacy is at stake. In line with keeping the procedure private, they expressed worries in case something went wrong at home or at workplace:

"“… it is shame to go back to the hospital; imagine people may be staring at you [circumcised man] that you were not circumcised at childhood… I mean it is shame for an adult man” (Man, 40 years, Christian)."

Other informants added that it is impossible for men working full-time to find time in their daily schedules to accommodate the circumcision procedure.

#### Interpersonal level (Interpersonal processes and personal groups: family, friends, and peers that provide social identity, support, and role definition)

The informants sensed that uncircumcised men might be neglected and disrespected in the community. Women elaborated that it is shameful, particularly for uncircumcised married men with children, to disclose their circumcision status. They considered that the surrounding community might know of the men’s circumcision status. One informant narrated:

"“Those who surround him will not understand him [uncircumcised man]. They will see him as an indigenous person. So he will keep it secret until when he experiences problems which require circumcision as a treatment” (Woman, 27 years, Christian)."

Another informant emphasised that there is uneasiness because everybody may know that a particular man has not been circumcised. Similarly, other informants stated that uncircumcised men might encounter shame when interacting with their peers. They voiced that, although seeking medical male circumcision might be for the best, the decision could be accompanied with negative consequences. One informant said:

"“They [uncircumcised] will feel guilty that, ‘I am now a grown up man. If I go to hospital, how are they going to perceive me?’ You see?… Perhaps, he may meet women who will laugh at him… (Man, 35 years, Christian)."

The informants reported that shame is obvious because instead of putting on a trouser, a freshly circumcised man wraps a cloth around his waist; sometimes he walks as if he has abscesses around the private parts. They thought that under such circumstances, one might skip going to work, and that colleagues could discover his circumcision status.

In addition, the informants stated that unlike circumcised men, uncircumcised men cannot date a woman or get married. Unlike the uncircumcised men, they felt that the circumcised men are more confident in initiating sexual relationships with women whom they love. One informant emphasised:

"“He [a circumcised man] has confidence… I mean if he wants to date a woman, he is always confident; not anxious like those who are uncircumcised. Uncircumcised men often suspect that if they date women, the women will see the penis, dislike it, disrespect them, and disclose their circumcision status to other women” (Man, 35 years, Christian)."

They reasoned that most women investigate the circumcision status of a man before they engage in a sexual relationship. Similarly, some men stated that most women prefer circumcised men.

#### Community level

##### Institutional factors (Rules, regulations, policies, and informal structures, which may constrain or promote recommended behaviours)

Informants described two types of male circumcision. These are medical male circumcision (MMC) and traditional male circumcision (TMC). They expressed confusion whether people should opt for MMC or TMC given the existing differences in terms of personnel and the cleanness of the procedure. They reported that a skilled person in the community (known as a Ngariba) performs TMC, while a trained health care provider in the hospital performs MMC. However, most informants expressed concerns about the cleanliness of the instruments in both types. They said that the ngariba performs the procedure by using shared unclean instruments and without painkillers. Although they realized that circumcision by the ngariba is cheaper, they noted that such an unclean environment could facilitate the transmission of infections. Nevertheless, they predicted that some people might choose the TMC and use their own cutting instruments to minimize the risks of infection. One informant stated:

"“Circumcision is performed at home by sharing knives and it is costless… people are now educated… those who prefer the traditional practice, they carry their own knives…” (Woman, 48 years, Christian)."

Although they said that some people might opt for a MMC because of hygiene, they expressed concern about the cleanliness of hospital instruments. Some of them suspected that people could acquire an infection from unsterilized and improper handling of the instruments. One informant cautioned:

"“Although you may be told the instruments were boiled [sterilized], you may find out that they are not clean. You do not know the truth! Perhaps, they [instruments] have been there for a long time and bacteria are there; then without knowing all that, a person is circumcised using those instruments” (Woman, 32 years, Christian)."

The informants encountered increased expenses in MMC as compared to TMC. They argued that the economically deprived parents might want to circumcise their children, but cannot afford the expense of the MMC. Based on experience, they predicted that many children are not undergoing MMC because their parents cannot afford the expenses of medicine, cotton wool, and gloves. Under such circumstances, they concluded that boys may get too old, and as they get older, the cost of circumcision increases. One informant reported:

"“To get circumcision service you need 15,000Tsh [US9$]… plus other costs; an older boy 20,000Tsh [US13$]; the oldest 30,000Tsh [US19$]. Don’t you see it is a budget for other things… school fees, house rent… The economic status may impose a barrier, and one can postpone the procedure, only to find out that the boy is getting older without being circumcised (Woman, 33 years, Muslim)."

Additionally, the majority of the informants were concerned about the cost involved in MMC. They stated that although the TMC is also costly, they found the cost to be negotiable. They also stated that the ceremonial expenses of TMC (jando) could be managed during the harvesting period.

Other informants reasoned that the issue of socio-economic status should not be a barrier to perform circumcision to the young boys. They proposed that the consequences of not circumcising might affect economic growth because men could get an HIV infection and die, leading to decreased manpower.

##### Community factors (social networks and norms, or standards, which exists as formal or informal among individuals, groups, and organizations)

Commonly, informants embraced male circumcision as a cultural practice through which awareness of responsibilities among young boys is enhanced. They stated that during circumcision they can teach young boys about what the community expects from them, and this becomes part of the culture. According to one informant:

"“During ‘jando’, the boys are taught about moral issues such as how to live with their parents; how to live with different tribes according to their cultural preferences…” (Man 37 years, Christian)."

The informants added that uncircumcised men might be prone to ignorance about their roles and expectations in the society. Unlike the circumcised men who know their roles from “jando,” the uncircumcised men lack that education. Thus, the uncircumcised men may be considered useless in the community as exemplified in the following quote:

"“A man walking on the street may see a kanga [a piece of cloth Tanzanian women cover themselves with] spread on the road. Those who have passed jando know what it means. They will look around trying to find out if there is a woman who is alone, about to deliver, and in need of help… they know what to do…; they will assist the woman according to what they were taught …” (Man, 37 years, Christian)."

Informants insisted that in some tribes uncircumcised men are grouped with immature boys; in other tribes they are warned that they will never get married and they are restricted from participating in cultural ceremonies. For example, one informant stated:

"“They may participate in the dowry receiving ceremony, but they will not be allowed to hold the dowry; they may be there but not in the front line” (Woman, 48 years, Christian)."

Most informants discussed how the community perceives uncircumcised men. They predicted that uncircumcised men might get old and die without having a sexual relationship with any woman because most women prefer circumcised men.

In contrast, some informants recalled that men from tribes that do not place a priority on male circumcision could marry from within their tribes. Other informants insisted that men from the tribes that do not circumcise should be regarded as normal because circumcision is not their cultural norm. In addition, they emphasised that being uncircumcised is not a reason of not fathering a child. One informant emphasised:

"“I would accept them the way they are because they are human beings… being uncircumcised does not mean that one is missing lots of things. Let us say, for example, to father a child; no, they can. They are just like other men…” (Man, 43 years, Christian)."

They stated that the man’s value is always there given that his circumcision status is kept secret. One woman emphasised that when a woman goes [has sex] with a man who is uncircumcised, she will never disclose that she had been with an uncircumcised man because friends will stare at her. Others noted that uncircumcised men might feel inferior in front of their colleagues and might harbour a sense of incompleteness, not speaking up as men, and always lagging behind because of that sense of incompleteness.

The informants reported that problems could arise when uncircumcised men move to big cities where male circumcision is commonly practiced. They said that uncircumcised men might be prone to stigma and discrimination from their peers in the cities who are circumcised. One informant stated that he would convince those who are not circumcised to be circumcised because he knows the benefits of male circumcision. Similarly, after marriage, some women said they might convince their uncircumcised partners to be circumcised.

##### Public policy (policies and laws that regulate or support healthy actions and practices for disease prevention, early detection, control, and management)

Informants expressed eagerness to have health policies that promote male circumcision. However, they cautioned that policy implementers should assess people’s cultural preferences. Most informants voiced the need of promoting the advantages of MMC particularly in regions with low male circumcision prevalence. From their experiences, they stated that the majority of the uncircumcised men reside in the villages. They stated that the government should integrate free male circumcision services within the routine childhood vaccination programmes and waive the circumcision fee in the hospitals. The following quote elaborated the idea:

"“This procedure should be given free of charge… perhaps it should be integrated in the routine childhood vaccination; once the baby boy is born he gets the service… there should be a record of all boys born in a certain month. Immediately when they get the third vaccination, they should be circumcised… circumcision should be part of the vaccination programme” (Woman, 33 years, Muslim)."

In addition, they emphasised on the need of education to promote male circumcision right after birth of a baby boy in the hospital. They believed that if mothers of the boys were educated after delivery and before discharge home, they might easily understand the importance of circumcision for the well being of their boys.

Informants stated that educating the mothers right after delivery could be a crucial time to aid in the decision to circumcise their sons. They suspected that when boys become older it would be difficult to point out who has been circumcised and who has not. They believed that it is important to promote male circumcision through public education, particularly in regions where this practice is not a priority. They also highlighted the importance of educating all men and women about the benefits of male circumcision. They spoke about use of culturally appropriate approaches to reach more people as well. One informant shared a desirable approach:

"“The older and respected men who are influential in the community should be contacted first. It is commonly understood that if you want to succeed in any programme, those men should be involved… when they are educated and demonstrate understanding, the next generation will understand through them” (Woman, 27 years, Christian)."

Others believed in a sensitization approach that takes place in stages, as emphasized in the following quote:

"Educate pupils from class five; in turn, they should sensitize their parents… Parents should educate their girls so that they can convince their boyfriends during sports’ participation to undergo circumcision (Man, 25 years, Christian)."

Overall, the informants are proponents of public education that promotes male circumcision among the community members.

## Discussion

The findings show diverse views regarding adult male circumcision. Knowledge, beliefs, perceptions and attitudes towards male circumcision seem to influence the acceptability of male circumcision among adults. Most informants expressed an increased awareness about the benefits of male circumcision in the prevention of sexually transmitted infections. Also, they related male circumcision with religious rituals and culture. Although they prioritized male circumcision as a health promoting practice, they expressed concerns including an interference with sexual desires and rescheduling of working timetable among adult men. However, they recognize the consequences of not performing circumcision. These consequences include shame, disrespect, stigma and lack of recognition in the community. They suggest that informal and formal policies should promote male circumcision and increase coverage of these services during childhood. In the following section, we discuss the major findings in line with the levels of SEM.

### Intrapersonal level

The demonstrated awareness towards male circumcision in prevention of STIs including HIV from infected woman to uninfected man is evidenced in other studies. In a Cochrane review article, MMC has proven to reduce the acquisition of HIV in heterosexual men by 38% to 66% over 24 months [33]. Similarly, in SSA, male circumcision has been associated with a significant decrease in risk of acquiring HIV infection among men [34]. Additionally, performance of male circumcision at childhood having a positive health impact is documented in previous studies. A study in Uganda suggested that pre-pubertal circumcision is more likely to protect against STIs, including HIV, as compared to post-pubertal circumcision [35]. However, a study in SSA suggests that circumcising men between 20 and 30 years of age can reduce adult HIV prevalence from 12% to 10% [36]. In the present study, the attitude that male circumcision may interfere with sexual desires among men highlights a potential obstacle towards the national effort of scaling up male circumcision among older men [19]. This could be the case in Iringa region, Tanzania, where only 24% of clients older than 20 years were served during the campaign of scaling up male circumcision [37]. Similarly, a previous study from Eastern and Southern Africa shows that attracting men over the age of 25 has proven more difficult than attracting young men to voluntary MMC [38]. The obstacle of getting older men to undergo circumcision may also be associated with working schedules that may disclose one’s circumcision status. On the other hand, the opinion that circumcision might interfere with sexual pleasure could contribute to denial of this service among adult men. However, a previous study in Uganda showed that male circumcision does not affect sexual functions [39]. Similarly, a few women experienced a decrease in sexual satisfaction after their partners were circumcised [40]. Nevertheless, for optimum health, it is suggested that infancy is the best time to perform circumcision [41]. Other studies have reported that neonatal circumcision may considerably reduce rates of STIs [42,43].

The belief that male circumcision holds religious and cultural implications is worth noting. The influence of religion can tremendously increase acceptability of male circumcision in the respective communities. In Tanzania, the majority of Muslims are circumcised [17], partly because Muslims hold a strong norm towards male circumcision that it is a confirmation of their relationship with God [5]. Also, the view in this study that male circumcision demonstrates adherence to religious rituals implies that all boys may be circumcised at childhood. Generally, the common determinants of male circumcision in most parts of the world are religion, ethnicity, and conformity to social norms [8,44].

### Interpersonal level

The argument that uncircumcised men may be convinced to undergo circumcision by their peers implies that peers may facilitate acceptability of male circumcision within their social networks. In addition, the existing norm about male circumcision and the interaction of peers in this community may automatically convince men from the tribes that do not circumcise to perform circumcision. This may be reflected in the diversity of ethnic groups among informants in this study and in the results of a previous study from this population, which showed that 96% of the participants were circumcised [32]. In Ghana, the majority of boys are circumcised because of the desire to conform, which is an important motivation for performing circumcision among them [5]. Similarly, the attitude of women disliking uncircumcised men may be due to the existing attitude of women’s preference of circumcised penis. Overall, peers and sexual partners may play a powerful role in influencing acceptability of male circumcision in the community where it is a strong norm.

### Community level

The expressed distrust about the cleanliness of instruments used both in MMC and TMC may seriously lead to deny of obtaining male circumcision services in either of the two setups. The distrust of instruments that are used in TMC is supported by a previous study in Tanzania where TMC is performed on groups of children using a non-sterile environment [17]. In a study done in Kenya, Lesotho, and Tanzania, circumcised male and female virgins were substantially more likely to be HIV infected compared to uncircumcised virgins [45], implying that HIV transmission could have occurred due to use of contaminated cutting instruments during circumcision. Similarly, in the Eastern Cape of South Africa traditional circumcision led to hospital admissions, amputations or mutilations and deaths which were directly related to the circumcision rituals [46]. The worry that unsterile instruments may be used in hospitals as well suggests that informants may avoid MMC even when it is promoted as the most safe service. However, a previous study in Kenya showed that MMC can be provided safely to adult men [3]. Similarly, from the recent male circumcision campaign in Tanzania, MMC resulted in a percentage of adverse events of less than 1% [37]. Another study in Tanzania proposed integration of traditional male circumcisers’ services into hospital services to enhance safety in the TMC set-up [47]. Overall, the perceived distrust of male circumcision services may be minimized if the circumcision is performed using sterile instruments and under medical supervision.

### Limitations

The findings are drawn from a small number of participants and from a limited geographical area. Also, according to a previous study [32], male circumcision is a strong norm in this population, which might have influenced the stated perceptions. Therefore, the study informants may not represent the general population. However, the findings provide in-depth knowledge regarding male circumcision in the limited local context, which is useful for programming at that level, but limited for national programming.

### Implications

#### Intrapersonal level

There is a need of increasing awareness among the community members that male circumcision reduces the risk of acquiring STIs including HIV infection from infected women to uninfected men. The Muslims and Christians should adhere to their religious rituals that facilitate male circumcision at childhood.

#### Interpersonal level

Peer influence may greatly contribute to health promotion activities including male circumcision in their respective communities provided that they have correct information.

#### Community level

The stakeholders from the Ministry of Health and Social Welfare (MoHSW) may use existing levels of health delivery services to ensure that male circumcision is safely done at minimal cost. Given the preference of TMC among some groups, the MoHSW should educate the traditional circumcisers to use clean cutting instruments in order to minimize chances of infection. The formal institutions such as schools and colleges should be used to disseminate education about the benefits of MMC. In the hospitals, the nurses may use the routine reproductive health education sessions to encourage mothers/parents to circumcise their boys at young age. Similarly, the older men who are recognised as influential in the community could be used to promote the safety of MMC in their respective communities.

## Conclusions

Generally, the study informants expressed diverse views regarding male circumcision. Although they are aware that male circumcision may prevent transmission of HIV and other sexually transmitted infections from infected women to uninfected men, they express worry towards circumcision at older age. Thus, integration of male circumcision services in the routine childhood vaccination program may increase coverage during the infancy period. However, further exploration of this possibility before implementing it is needed. To increase acceptability of male circumcision, there is a need to monitor the safety of the procedure in both hospital and traditional settings. Overall, the findings from this investigation provide contextual understanding that may assist in scaling-up male circumcision in Tanzania.

## Competing interests

The authors declare that they have no competing interests.

## Authors’ contributions

EAMT conceived the study, participated in study design, coordinated data collection, carried out analysis and drafted the manuscript. JF conceived the study, participated in the study design, coordinated and participated in data collection and critically reviewed the manuscript. DK conceived the study, participated in the study design, data analysis and critically reviewed the manuscript. PM, MB and ES conceived the study, were involved in the study design and critically reviewed the manuscript. All authors read and approved the final manuscript.

## Pre-publication history

The pre-publication history for this paper can be accessed here:

http://www.biomedcentral.com/1471-2458/12/529/prepub
